# Right hepatectomy for a detoured left hepatic artery in hilar cholangiocarcinoma—report of a rare but rational resection

**DOI:** 10.1186/s12957-016-1045-8

**Published:** 2016-11-16

**Authors:** Chun-Yi Tsai, Nobuyuki Watanabe, Tomoki Ebata, Takashi Mizuno, Yuzuru Kamei, Masato Nagino

**Affiliations:** 1 Division of Surgical Oncology, Department of Surgery, Nagoya University Graduate School of Medicine, 65 Tsurumai-cho, Showa-ku, Nagoya, 466-8550 Japan; 2 Department of Plastic and Reconstructive Surgery, Nagoya University Graduate School of Medicine, Nagoya, Japan

**Keywords:** Perihilar cholangiocarcinoma, Simultaneous vascular resection, Hepatic artery resection, Variant hepatic artery

## Abstract

**Background:**

Curative hepatectomy with bile duct resection is the treatment for perihilar cholangiocarcinoma. A locally advanced tumor necessitates hepatectomy with simultaneous vascular resection, and reconstruction remains an obstacle for surgeons. Studies have focused on the variations of hepatic arteries. Nevertheless, the anatomical alignment of the portal veins, bile ducts, and hepatic arteries are equally critical in surgical planning of curative resection for advanced tumors. We have reported promising outcomes of hepatectomy with simultaneous resection and reconstruction of the hepatic artery. With respect to the type of surgery, most patients undergo left hepatectomy with right hepatic artery resection and reconstruction in contrast to right hepatectomy with left hepatic artery resection and reconstruction. We present two patients who showed detoured left hepatic arteries that were invaded by the perihilar tumors.

**Case presentation:**

A 78-year-old man who presented with epigastric pain and abnormal liver function was referred to our clinic for further examination. Serial examination resulted in the diagnosis of Bismuth type II hilar cholangiocarcinoma. The left hepatic artery ran a detoured course and was invaded by the tumor. The second patient was a 76-year-old woman who presented with jaundice and the Bismuth type II hilar cholangiocarcinoma. The left hepatic artery was along the right-lateral position of the left portal vein and was invaded by the tumor. The variant anatomical relationship of the vessel was identified preoperatively in both patients, and they underwent right hepatectomy with concomitant left hepatic artery resection and reconstruction without any major complications or recurrence.

**Conclusions:**

The largely biased selection of patients is based on the following anatomical relationship: the left hepatic artery usually runs left lateral to the portal vein, which spares invasion by the perihilar cholangiocarcinoma. On the contrary, the right hepatic artery mostly runs behind the bile duct and is invaded by the tumor. This aforementioned anatomy is one of the reasons for the relatively rare left hepatic artery resections and reconstructions in right hepatectomies. By meticulous preoperative evaluation with images, we identify the anatomical variation and performed right hepatectomy with concomitant left hepatic artery resection and reconstruction without any major complications and mortalities.

## Background

Perihilar cholangiocarcinoma is the most prevalent biliary malignancy and accounts for approximately 65% of biliary malignancy cases. Radical resections such as hepatectomy plus extrahepatic bile duct resection remains the only curative treatment. The portal veins and hepatic arteries were frequently invaded in locally advanced perihilar cholangiocarcinomas because of their anatomical orientation. Simultaneous vascular resection and reconstruction is warranted to achieve curative resection for such cases. The concomitant resection and reconstruction of the contralateral hepatic artery remains an obstacle [1]. We adopted aggressive surgical resection for perihilar cholangiocarcinoma and accumulated a large number of hepatectomies with simultaneous resection and reconstruction of the portal vein and/or hepatic artery with acceptable results [2]. Regarding the types of operation among these patients, the right hepatectomy along with left hepatic artery resection and reconstruction is performed less often than the left hepatectomy along with right hepatic artery resection and reconstruction. The above observation results from the anatomical relationship of bile duct, portal vein, and hepatic arteries at the perihilar area, which spared the left hepatic artery from invasion by the advanced perihilar cholangiocarcinoma. In the following cases, we demonstrate that we were able to identify the detoured left hepatic artery preoperatively and successfully achieve the rare type of operation as per our planning.

## Case presentation

**Fig. 1:**
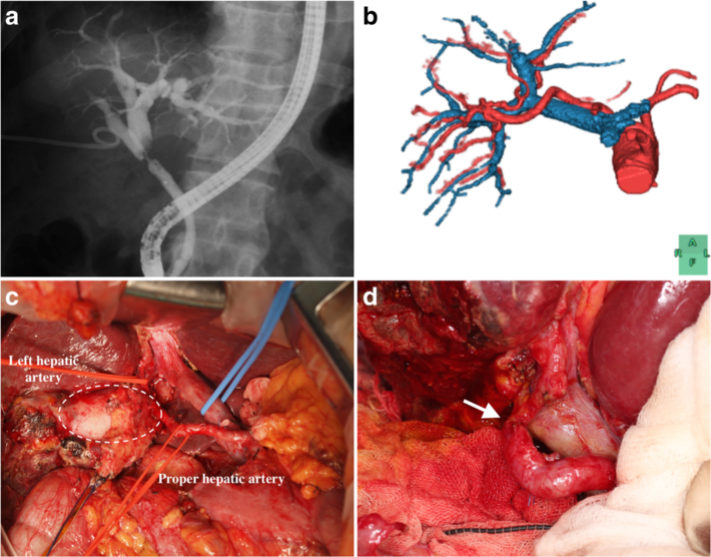
**a** The cholangiography shows a Bismuth type II perihilar cholangiocarcinoma. **b** The MDCT-generated arteriogram and portogram disclose the “detoured” left hepatic artery. **c** The left hepatic artery and the hepatic proper artery were slung with *red tapes. Dashed lines* indicate the tumor location. **d** End-to-end reconstruction between the left hepatic artery and the hepatic proper artery. The *arrow* indicates the anastomosis

A 78-year-old man who presented with epigastric pain and elevated liver functional enzymes was referred to our clinic for further examination. Endoscopic retrograde cholangiography (ERC) revealed biliary obstruction at the confluence of the left and right anterior bile ducts, resulting in the diagnosis of Bismuth type II hilar cholangiocarcinoma (Fig. 1a). An arteriogram generated by multidetector-row computed tomography (MDCT) showed that the proper hepatic artery ran transversely and right laterally to the right side of the hepatoduodenal ligament, which then branched towards the left hepatic artery (Fig. 1b). Invasion of the hepatic artery was highly suspected. Four weeks after right portal vein embolization, right hepatectomy and caudate lobectomy combined with left hepatic artery resection were performed. The hepatic artery was reconstructed by direct end-to-end anastomosis (Fig. 1c, d). The tumor was a moderately differentiated adenocarcinoma with microscopic invasion of the resected hepatic artery but without any lymph node metastasis. The patient was discharged from the hospital on day 24 without major complications and has since enjoyed a normal social life without any recurrence for 26 months.

**Fig. 2:**
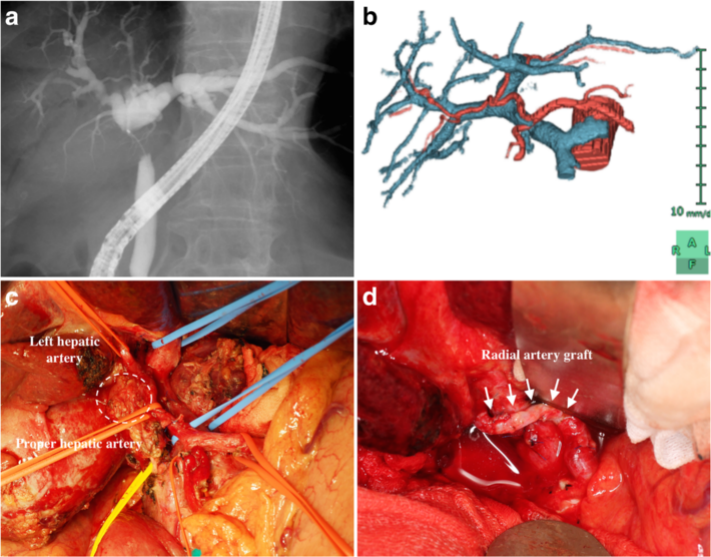
**a** The cholangiography shows a Bismuth type II perihilar cholangiocarcinoma. **b** The MDCT-generated arteriogram and portogram disclose the relatively right-sided cruising hepatic proper artery and left hepatic artery. **c** The tumor invaded the bifurcation of both the hepatic arteries and the portal veins. **d** An interpositional radial arterial graft for reconstruction of the left hepatic artery. *Arrows* indicate the radial arterial graft

The second patient was a 76-year-old woman who presented with jaundice. MDCT and ERC with biopsy confirmed the diagnosis of Bismuth type II hilar cholangiocarcinoma (Fig. 2a). The reconstructed arteriogram showed the proper hepatic artery ran transversely and bifurcated into the right and left hepatic arteries just behind the perihilar tumor, and the left hepatic artery ran right lateral to the left portal vein (Fig. 2b). MDCT showed possible invasion of both the hepatic artery and the portal bifurcation. Three weeks after right portal vein embolization, right hemihepatectomy and caudate lobectomy combined with simultaneous resection of the hepatic artery and portal vein were performed. The left hepatic artery was reconstructed using an interposition radial artery graft (Fig. 2c, d). The left portal vein was reconstructed with direct end-to-end anastomosis. The tumor was a poorly differentiated adenocarcinoma without lymph node metastasis; however, microscopic invasion of the resected hepatic artery and portal vein was found. After control of the biliary fistula, the patient was discharged from the hospital on day 46 and is alive without any recurrence 7 months after surgery. Our practice and surgery followed the guidelines of the Japanese Hepato-Biliary-Pancreatic Association. Written consent forms for publication of this manuscript are available from both patients.

## Conclusions

Perihilar cholangiocarcinoma is the most challenging malignant tumors for surgeons, owing to its complicated perioperative management and relatively unfavorable prognosis. Hepatectomy with dissection of regional lymph nodes including areas around common hepatic artery, hepatoduodenal ligament, and retropancreatic region (group 8, 12, and 13 according to the Japanese classification) plus extrahepatic bile duct resection is our standard treatment for Bismuth type I or II tumors [3]. In locally advanced tumors, vascular invasion, more commonly hepatic arterial invasion, remains the most serious obstacle in the resection of this disease. Owing to the refinement of surgical skills and the increase in number of cases, hepatectomy with concomitant contralateral hepatic artery resection and reconstruction has recently been conducted in leading centers [1, 4]. In our study in 2010, which is the largest series in a single institute, 49 of 50 patients underwent left-sided hepatectomy with right hepatic artery resection. Meanwhile, the one remaining patient underwent right-sided hepatectomy with left hepatic artery resection [5]. The variations of the hepatic artery and its branches have been reported and reviewed for decades [6, 7]; however, their alignments within the perihilar area were not mentioned. Regardless of their variant branches, the relative locations and alignments among the portal veins, hepatic arteries, and bile ducts are equally critical in the planning of surgery for advanced perihilar cholangiocarcinomas. Our largely biased selection of left-sided hepatectomy was based on the following anatomical reasons: In the vast majority of patients, the right hepatic artery runs behind the common hepatic duct, and thus, the Bismuth type II or the left-side predominant perihilar cholangiocarcinoma easily involves the right hepatic artery. By contrast, the left hepatic artery usually runs along the left-most portion of the hepatoduodenal ligament; therefore, the Bismuth type II or the right-side predominant tumor rarely involves the left hepatic artery. With respect to our two patients, their hepatic arteries were along the relatively right aspect of the portal vein and were invaded by the tumors.

In our institute, preoperative MDCT has been applied to assess invasion of the hepatic artery by the tumor [8]. The image could also provide vital information about the anatomical relationship of the vessels and tumors. With meticulous evaluation of images, the rare anatomical relationship among the hepatic arteries, portal veins, and bile ducts at the perihilar area could be identified preoperatively. Surgeons can elaborate feasible planning for these patients. To our knowledge, this is the first report of a successful combined resection for such a rare case. Right hepatectomy with left hepatic artery resection and reconstruction may be an extended but rational procedure that can offer promising results for patients with advanced perihilar cholangiocarcinoma possessing a detoured left hepatic artery.

## Abbreviations

ERC: Endoscopic retrograde cholangiography
MDCT: Multidetector-row computed tomography
